# Epigenetic and Post-Translational Regulation of Schlafen Family Expression and Their Differential Methods of Regulating Proteins

**DOI:** 10.3390/ijms262211058

**Published:** 2025-11-15

**Authors:** Odele Rajpathy, Emilie E. Vomhof-DeKrey

**Affiliations:** 1 Department of Pathology, School of Medicine and the Health Sciences, University of North Dakota, Grand Forks, ND 58202, USA; odele.rajpathy@und.edu; 2 Department of Surgery, School of Medicine and the Health Sciences, University of North Dakota, Grand Forks, ND 58202, USA; 3 Department of Biomedical Sciences, School of Medicine and the Health Sciences, University of North Dakota, Grand Forks, ND 58202, USA

**Keywords:** Schlafen proteins, SLFN, Slfn, epigenetic regulation, gene expression, post-transcriptional regulation, cell proliferation, differentiation, cancer

## Abstract

Schlafen (SLFN) proteins are a unique and emerging yet incompletely understood family that have primarily been investigated for their putative roles in immunological responses, cell proliferation, and non-malignant cell differentiation. Increasingly, SLFNs have been implicated in diverse biological and pathological contexts, including cancers, viral replication, embryonic lethality, meiotic drive, and inflammatory bowel diseases, where they may be either genetically upregulated or downregulated. In recent years, novel insights into their functional similarities and distinctive particularities have intensified interest in this gene family. This review critically evaluates the biology of SLFN proteins with a specific focus on the epigenetic regulation of their expression and the differential methods by which they regulate downstream proteins. Evidence indicates that SLFNs act not only as regulators of transcription but also as modulators of gene expression through post-transcriptional modifications and epigenetic mechanisms, which demonstrate their multifaceted and context-dependent activity across disease models. By consolidating these findings, this review brings to light the physiological and pathological significance of SLFNs and identifies key gaps in understanding their epigenetic control and mechanistic diversity, thereby offering directions for future research.

## 1. Introduction

### 1.1. Origin and Derivation

The Schlafen (Slfn) genes were first discovered and identified in mice in 1998 by Schwarz et al. during a screening for genes involved in thymocyte maturation. These genes produced proteins that could be divided into three different groups according to their length, which sequentially came to be known as the murine Slfn proteins [1].

An initial study of the Slfn family genes revealed a significant increase in their expression throughout thymocyte development. During thymocyte maturation, Slfn1 and Slfn2 were both upregulated in expression when comparing the double-positive early thymocyte maturation stage (positive for CD4 and CD8) and the single-positive final thymocyte maturation stage of development; however, Slfn4 was downregulated. The study broadly noted that the most prominent attribute of this gene family was its effect on cell growth and progression throughout the cell cycle. Upon experimentation, it was discovered that Slfn1 stimulation resulted in a thymocyte cell cycle arrest at the G0/G1 stage, and they were unable to initiate DNA synthesis, which closely resembled cells being put to “rest”. Furthermore, by inhibiting Slfn1 gene expression, the growth inhibition could be reversed. As a result, the family was given the German name Schlafen, which means “to slumber” [2].

**Figure 1:**
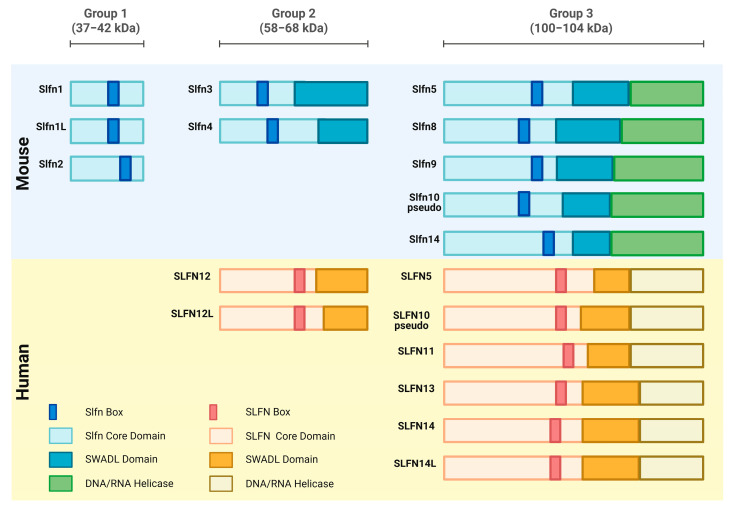
Diagram illustrating the relative sizes of Schlafen subgroups and a comparison of the Schlafen family members’ linear domain structures. The Schlafens domains in humans and mice are shown. Created in https://BioRender.com.

Since then, subsequent studies have focused on deciphering the genetic architecture of Slfns and have demonstrated their expression in a variety of vertebrates, including humans. Summarization of the Schlafen family now includes 10 murine genes Slfn1, Slfn1L, Slfn2, Slfn3, Slfn4, Slfn5, Slfn8, Slfn9, Slfn10 pseudogene, and Slfn14 and 8 human genes, SLFN5, SLFN 10 pseudo, SLFN11, SLFN12, SLFN12L, SLFN13, SLFN14, SLFN14L expressed by humans [3,4] (Figure 1).

### 1.2. Schlafen Gene Characteristics and Subcellular Localization

The human Schlafen genes are primarily localized on chromosome 17, while the mouse Schlafen genes are found on chromosome 11. Orthologs, defined as coexisting genes in different species, were identified between murine and human Schlafen family members. SLFN5 and Slfn5, along with SLFN14 and Slfn14 function, as direct orthologs between humans and mice. Murine Slfn3 and Slfn4 share significant homogeneity with human SLFN12 and SLFN12L family members. The diversity of Schlafen expression across species extends throughout all mammalian genomes with substantial coverage [5]. Schlafens were also interestingly found in one amphibian (*Xenopus laevis*) and one fish species (*Callorhincuys milii*), but not in other non-mammalian species with extensive genome coverage [5]. Viruses have also been studied to express a Schlafen ortholog known as v-slfn, which is associated with the regulation of viral replication and plays an important role in decreasing viral susceptibility to host defense mechanisms [6].

As in the case of any protein, Schlafen proteins possess a strong structure–function relationship. The molecular structure of the Schlafen family members is unique and bears no resemblance to other existing proteins. In the Schlafen family genes, there is a Slfn-box domain, which acts as a genetic feature that distinguishes it from other protein structures [7] (Figure 1).

Based on differing lengths and molecular structures with a range of 337 to 910 amino acids, the Schlafens are classified into three primary subgroups. Group I members consisting of Slfn1, Slfn1L, and Slfn2, possess a molecular weight between 37 and 42 kDa and are the shortest in length. Group II members consisting of Slfn3, Slfn4, SLFN12, and SLFN12L possess a molecular weight between 58 and 68 kDa and are intermediate in length while Group III members, consisting of Slfn5, Slfn8, Slfn9, Slfn10pseudo, Slfn14, SLFN5, SLFN10 pseudo, SLFN11, SLFN13, and SLFN14, and SLFN14L, possess a molecular weight between 100 and 104 kDa and are the longest in length. Schlafens also contain additional domains such as a divergent AAA domain and a domain known as the “SWADL” domain, which is specific to Group I and Group II Schlafen members [3]. Group III members contain a supplementary C-terminal domain that is homologous to the DNA/RNA helicases I family and harbors a nuclear targeting sequence, which suggests that long Schlafens act predominantly within the nucleus [8]. This nuclear localization signal is not found in the short and intermediate Schlafens, and they predominantly operate in the cytoplasm (Figure 1) [9,10].

## 2. Schlafen Function in Mice and Humans

### 2.1. Role in Immune Cell Differentiation

One of the central functions of the Schlafen genes lies in the immune response when activated by stimulators. During the maturation stages of the thymus, studies have reported upregulation in Slfn1, Slfn2, and Slfn3 during the gradual transition from the CD4^+^ CD8^+^ double-positive stage to the single-positive mature stage. Conversely, a downregulation is seen in Slfn1 and Slfn2 when T-cells are activated [1]. Upon activation by inflammation inducers, such as IFNα, lipopolysaccharide (LPS), and bleomycin treatment, and other cells demonstrating immune functions, like melanocytes [11], microglia [12] and alveolar MLE-12 cells [13], respectively, expressed an upregulation of Slfn1 and Slfn2. In contrast, there is a downregulation of Slfn4 during macrophage differentiation but an upregulation during macrophage activation. Mouse Slfn4 upregulates upon treatment with IFNα in melanocytes, LPS in microglia, and bleomycin in alveolar MLE-12 cells. Naive T cells are actively maintained in a quiescent state that promotes their survival and persistence, wherein Slfn2 plays an influential role in the growth and differentiation of mouse hematopoietic cells, which is critical for T cell quiescence [14].

### 2.2. Role in Malignant Cell Differentiation

**Figure 2:**
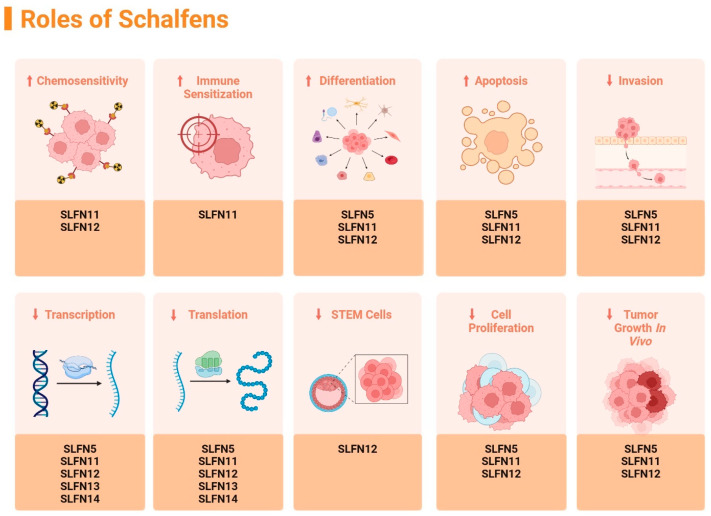
Summary of the identified effects of different Schlafens on malignant cell biology, figure adapted from reference [10]. (↑ = increase, ↓ = decrease). Created in https://BioRender.com.

The Schlafen family members have been extensively studied in relation to their effect on malignant cell differentiation. These proteins all exhibit differential upregulation and downregulation in cancers such as breast cancer, lung squamous carcinoma, prostate cancer, and rectal carcinoma. Human SLFN5, SLFN11, SLFN12, SLFN13, and SLFN14 have been strongly linked to being positive markers when diagnosing malignancy. As shown in Figure 2, human SLFN family members play beneficial roles in increasing chemosensitivity, decreasing in vivo tumor growth, decreasing cell proliferation, decreasing stem cell growth, decreasing expression in translation and transcription, decreasing invasion of cancerous cells, increasing induced apoptosis of malignant cells, and increasing cell differentiation [10].

### 2.3. Role in Viral Replication

Viral slfn (v-slfn) was discovered in the camelpox virus and other orthopoxviruses by Gubse et al. in 2007 [15]. Reported results suggest that invading viruses may utilize v-slfn to dampen the host immune responses, thereby promoting immunodeficiency. However, other studies report that the Slfn11 impairs the replication of flaviviruses, including West Nile virus, dengue virus, and Zika virus, although replication of single-stranded negative RNA viruses was not affected [16].

## 3. Emerging Importance of Epigenetic Regulation in Schlafen-Mediated Immunity and Cancer

Although SLFN proteins are increasingly recognized for their diverse roles in immune regulation, viral restriction, and cancer biology, their epigenetic regulation remains very poorly understood. Foundational studies established SLFNs as modulators of thymocyte differentiation and immune activation [1,2]. More recent work linked them to tumor suppression, DNA damage response, and antiviral defense [7,17]. Yet, evidence now shows that epigenetic silencing via DNA methylation, deacetylation, and post-translational phosphorylation can downregulate key members like SLFN11 and SLFN12, driving chemoresistance and poor outcomes in cancers such as gastric, colorectal, and ovarian tumors [18,19,20]. Reactivating SLFN11 via HDAC inhibitors restores cancer cell sensitivity to DNA-damaging agents [21,22], while SLFN12 influences immune regulation and chemotherapy response in triple-negative breast cancer [23]. Dysregulated SLFNs are also linked to autoimmune diseases such as Multiple Sclerosis and Hashimoto’s Thyroiditis [24,25]. This review is therefore timely and necessary, as it unifies fragmented findings in existing literature to delve into how epigenetic modulation of SLFN genes holds strong therapeutic potential in the clinical setting, which can be harnessed for targeted interventions in cancer, immune, and viral diseases.

## 4. Role of Regulatory Influence on the Expression of Schlafen Family Members

The effects of Schlafen family member downregulation or loss on the expression of other family members are poorly understood. Hence, this was further studied by utilizing Slfn3 knockout mice to investigate how the loss of Slfn3 differentially affects the expression of other Slfn family members in the spleen, thymus, and intestinal mucosa. The deletion of Slfn3 had the greatest impact on Slfn8 and Slfn9, with decreased expression in the ileal mucosa, while there was an increased expression for both Slfns in the splenic and thymic tissues [26]. The differential Slfn family member expression observed in response to Slfn3 deletion may point to a potential feedback regulatory mechanism within the Slfn family, which sets the Schlafen protein family apart from other protein families. Considering that Slfn8 and Slfn9 are located in the nucleus, whereas Slfn3 is in the cytoplasm, it is interesting that the loss of Slfn3 might impact the RNA expression levels of nuclear Slfns. This observation may be further contextualized by recent studies on SLFN12 demonstrating its interactions with PD3DA and RNase activity [27,28,29,30,31,32], which will be discussed in greater detail in the phosphorylation section below. Schlafen family members’ expression changes in Slfn3KO mice may be altered to compensate for the loss of Slfn3 [33].

In addition, recent work has also investigated the role of SLFN12 in regulating other SLFN family members in the context of triple-negative breast cancer (TNBC) and chemotherapy response. One study demonstrated that interferon-α2 (IFN-α2) treatment upregulates multiple SLFN family members, including SLFN12, in TNBC cells, reducing cell viability and suggesting a complex intra-regulation between family members [23]. Importantly, SLFN12 knockdown revealed compensatory regulation by other SLFN members under IFN-α2 and chemotherapy treatment, which correlates with the Slfn3KO mice studies and suggests an intra-regulation of SLFN family expression [34].

## 5. Regulation of Schlafen Family Members by Epigenetic Modifications

### 5.1. Brief Overview of Epigenetic Modifications

The term “Epigenetics” is used to refer to heritable alterations that are not due to variations in DNA sequence. Rather, epigenetic modifications such as DNA methylation and histone modification alter DNA accessibility and chromatin structure, thereby regulating patterns of gene expression. These processes are crucial to the normal development and differentiation of distinct cell lineages in the adult organism [35]. The transcriptional effects of epigenetic regulation are multidimensional as they not only function as an on- or off-regulation but also include the moderation of the kinetics and robustness, maintenance of transcriptional status, and responsiveness or unresponsiveness to external stimuli of transcription. Accordingly, epigenetic modifications coordinate various aspects of immune cell biological events, including maintenance of differentiation, effector functions, and plasticity. Epigenetic modifications were initially recognized as crucial regulators of cell differentiation and development, but research over the last few years has identified a novel and dynamic role for epigenetics in regulating Schlafen family members.

Although research on epigenetic modifications affecting Schlafen protein function is still emerging, growing evidence is bringing to light important connections between histone modification mechanisms and the regulation of Schlafen family members. These insights offer promising new directions for understanding and improving chemosensitivity in cancer therapy [36]. Histone proteins undergo post-translational modifications in different ways, which impact their interactions with DNA. There are at least nine different kinds of histone modifications discovered but the most well-known types of modification include acetylation, methylation, phosphorylation, and ubiquitylation [37].

### 5.2. Acetylation

Acetylation has been one of the most extensively investigated histone modifications due to its significant impact on transcriptional regulation. Positively charged lysine residues in histones are neutralized by acetylation, which reduces the interaction between histones and negatively charged DNA. This charged neutralization diminishes the binding between histone and DNA, thereby allowing transcription factors to bind and markedly increasing gene expression [38]. Histone acetylation regulates numerous physiological processes, including cellular differentiation, DNA replication and repair, nuclear import, neural repression, the cell cycle, cell proliferation, and cell death [39].

Prior studies have identified SLFN11 expression as a major determinant of cancer cell sensitivity to DNA-damaging chemotherapeutic agents across several cancers. SLFN11 expression correlates positively with susceptibility to cytotoxic treatments, such as topoisomerase inhibitors. For instance, a study on the cell surface receptor CD47, which protects non-malignant cells from genotoxic stress caused by chemotherapy but sensitizes targeted tumors, revealed that SLFN11 was consistently downregulated in CD47-deficient cells. This study reported a direct correlation between a decrease in acetylation of the *SLFN11* promoter gene region and a decrease in SLFN11 expression [40].

Further research confirmed that the epigenetic inactivation of SLFN11 leads to resistance against induced malignant cell genotoxicity. They demonstrated that this resistance could be overcome by deacetylase inhibitors, which function to derepress SLFN11 expression, thereby sensitizing tumor cells to chemotherapeutic mechanisms [21]. The fluctuation of SLFN11 levels through acetylation directly influences the effectiveness of DNA-damaging therapies, with loss of acetylation fostering drug resistance and increased acetylation restoring chemosensitivity. Applying this SLFN11-dependent epigenetic mechanism in a clinical setting could suggest using a therapeutic combination of DNA-targeted agents and deacetylase inhibitors to overcome drug resistance.

Results reported by a study investigating small cell lung cancer (SCLC) cells demonstrated that the histone deacetylase inhibitor FK228 can induce the re-expression of SLFN11. This reactivation is primarily achieved by promoting histone acetylation at the promoter region of the *SLFN11* gene, which subsequently leads to a significant reduction in DNA methylation. The increase in histone acetylation weakens the interaction between histones and DNA, facilitating the binding of transcription factors and thereby enhancing SLFN11 expression [22].

Similarly, another study investigating the role of histone acetylation in regulating SLFN11 expression in germinal center B cells (GCBs) during B cell development reported that FK228 could induce SLFN11 expression by promoting histone acetylation at the SLFN11 promoter. Treatment with epigenetic modifiers such as the HDAC inhibitor, Panobinostat, significantly upregulated SLFN11 expression across various GCB-derived lymphoma cell lines, indicating that histone acetylation can reverse the suppression of SLFN11 [41].

Elevated SLFN11 enhances the cell’s DNA damage response, promoting apoptosis and genomic stability, and plays a key role in processes such as lymphocyte differentiation. Conversely, reduced acetylation diminishes SLFN11 expression, impairing DNA repair pathways and allowing cancer cells to resist genotoxic stress and continue proliferating. Importantly, these fluctuations can be therapeutically targeted: deacetylase inhibitors restore SLFN11 expression, reactivating its tumor-suppressive functions and sensitizing resistant cancer cells to DNA-damaging treatments (Table 1).

### 5.3. Methylation

Methylation involves the addition of a methyl group to the lysine or arginine amino acids on histones H3 and H4 and results in varying effects on transcription. Arginine methylation encourages transcriptional activation, whereas lysine methylation can influence either transcriptional activation or repression depending on the methylation position. This adaptability may be explained by the fact that, unlike acetylation, methylation does not affect the histone charge or the direct connections between histones and DNA [42].

SLFN11 expression is severely diminished when methylated in contrast to acetylation. The putative transcriptional inactivation of the SLFN11 gene at the RNA and protein levels was assessed in patients with ovarian and non-small cell lung cancer, and it was reported to have found a poor response to cancer treatments, cisplatin and carboplatin, suggesting a mechanistic pathway that inactivates SLFN11 expression when it is hypermethylated. This was further confirmed with in vitro silencing of SLFN11 gene expression, which increased resistance to cisplatin and carboplatin treatments [43].

In small cell lung cancer (SCLC) cells, SLFN11 expression is strongly suppressed by promoter methylation. Treatment with the HDAC inhibitors FK228 and SAHA reduced this methylation in a dose-dependent manner, restoring SLFN11 expression and sensitizing cells to topotecan. Clinical analysis further revealed that most SCLC specimens exhibited SLFN11 promoter methylation [22].

SLFN11 is often methylated in human stomach cancer, and experimental findings demonstrated that SLFN11 functions as a tumor suppressor in human gastric cancer, and that methylation of SLFN11 may act as a marker for cisplatin resistance in stomach cancer cells. Methylation of SLFN11 was also significantly associated with tumor size (*p* < 0.05). SLFN11 suppressed gastric cancer growth both in vitro and in vivo, which suggested an enhanced ability of cisplatin to induce S-phase arrest and apoptosis in gastric cancer cells [19].

Another study that examined SLFN11 in colorectal cancer patients’ samples recorded SLFN11 to be methylated in 55.47% (71/128) of the colorectal cancer patient samples. Unmethylated SLFN11 has also been found to stably suppress colorectal cancer proliferation and sensitize colorectal cancer cells to cisplatin treatment, as in the case of ovarian cancer cells as well [18]. Thereby, SLFN11 functions as a tumor suppressor in colorectal cancer, and its methylation status may indicate cisplatin resistance.

In bladder cancer cells, SLFN11 expression was noted to be significantly reduced compared to normal tissue, where this downregulation was strongly associated with increased methylation of its gene locus. This methylation was inversely correlated with SLFN11 mRNA levels, which suggests that epigenetic silencing contributes to decreased protein expression in cancer cells. Experimental data from this study also established that treatment with demethylating agents, such as 5-Aza, can partially restore SLFN11 expression, indicating methylation as a reversible regulatory mechanism [44].

In another study examining SLFN11 as a predictor of platinum sensitivity in ovarian cancers, including high-grade serous carcinoma and clear cell carcinoma independent of BRCA1/2 mutation status, results demonstrated that promoter hypermethylation can epigenetically silence SLFN11 expression, contributing to chemotherapy resistance [20]. Physical exercise can interestingly modify DNA methylation patterns in postmenopausal women with poor glycemic control. The SLFN12 gene was hypermethylated in prediabetic women and became downregulated, but exercise reduced its methylation, leading to upregulation of SLFN12. This normalization of SLFN12 methylation was associated with improved glucose and lead (Pb) levels [45].

A study involving isolated CD4^+^ and CD8^+^ T cells from multiple sclerosis (MS) patients indicated regions of hypermethylation in SLFN12 owing to an overall decrease in SLFN12 expression in whole blood samples of multiple sclerosis patients when compared to controls. This demonstrated a comparable reaction of increased SLFN12 methylation and corresponding gene downregulation. These regions may influence disease etiology and shed light on risk factors for MS [24] as SLFN12 is a biologically plausible gene of interest for MS since there is substantial evidence from clinical observations and genetic studies that MS pathology is driven by T cells and that IFN beta type I, which induce expression of SLFN genes is an authorized treatment for MS [46].

In Hashimoto’s Thyroiditis patients, the SLFN12 promoter exhibited extreme hypomethylation, resulting in significantly elevated mRNA expression that was inversely correlated with methylation levels [25].

Interestingly, existing literature suggests that hypermethylation-mediated silencing of SLFN11 and SLFN12 disrupts DNA damage recognition, impairs apoptosis, and promotes chemoresistance across multiple cancers, allowing malignant cells to persist despite therapeutic intervention. Whereas demethylation or hypomethylation can restore tumor-suppressive activity, re-sensitizing cancer cells to DNA-damaging agents and reinstating genomic surveillance mechanisms. Beyond oncology, altered SLFN12 methylation in autoimmune diseases such as multiple sclerosis and Hashimoto’s thyroiditis highlights its broader role in immune homeostasis, where methylation shifts may drive either immune suppression or hyperactivation. Collectively, these fluctuations illustrate how methylation acts as a molecular switch that dynamically governs SLFN gene function, balancing cellular proliferation, immune regulation, and therapeutic sensitivity in both physiological and pathological contexts (Table 1).

### 5.4. Phosphorylation—Epigenetic and Post-Translation Modifications

Histone phosphorylation involves the addition of a negative phosphate group to histone proteins, which are involved in chromatin compaction and the regulation of chromatin structure and function during mitosis. Histone phosphorylation is a critical intermediate step in chromosome condensation during cell division, transcriptional regulation, and DNA damage repair [47].

Although this paper primarily focuses on the epigenetic regulation of SLFN genes, we also included a discussion of post-translational modifications in addition to the epigenetic modifications of phosphorylation, due to their crucial role in modulating SLFN protein function after translation. Including post-translational phosphorylation provides a more comprehensive understanding of how SLFN activity is controlled beyond transcriptional and chromatin-level mechanisms to influence SLFN-mediated responses to DNA damage, cell proliferation, and tumor sensitivity to therapy.

The mutation studies undertaken by Malone et al. investigated the impact of the phosphatase inhibitor okadaic acid on the inhibition of SLFN11. This study strongly supported the notion that SLFN11 activation requires dephosphorylation of the gene [48]. Inversely, phosphorylation of the gene inhibited the repressive cell proliferative functions of SLFN11, which normally enabled tumor shrinkage in targeted cancer therapy. Further research identified phosphorylation sites S219, T230, and S753 as key regulators that inhibit SLFN11’s ribonuclease activity. In Sus scrofa (wild boar), SLFN11 is phosphorylated at S214, which is located within a positively charged RNA-binding region. It was postulated that phosphorylation at S214 likely neutralizes this charge, impairing RNA binding and RNase function [49]. Additional structural analyses further revealed that phosphorylation at S753 within the helicase domain also impairs SLFN11’s ability to bind single-stranded DNA and cleave tRNA, as demonstrated by loss of function in phospho-mimetic mutants [50].

Another study discovered that SLFN11’s ability to sensitize cells to DNA-damaging agents is tightly regulated by its phosphorylation of SLFN11 protein. Dephosphorylation enhances SLFN11’s binding to single-stranded DNA, enabling it to block DNA replication and increase drug sensitivity. Conversely, phosphorylation or mutations that mimic constant phosphorylation disrupt this binding and abolish SLFN11’s function. In the study, protein phosphatase 2A was identified as a key regulator that interacts with SLFN11, likely controlling its dephosphorylation and activity [51].

SLFN12 RNase activity’s regulation by phosphorylation and its interaction with PDE3A, a phosphodiesterase enzyme, has also recently been studied. Binding to PDE3A triggers dephosphorylation of SLFN12 at key sites (S368, S573), activating its RNase function and upregulating the protein [31,52]. Recent studies have provided significant mechanistic and translational insights into the regulation of SLFN12 by PDE3A modulators, where early work identified PDE3A modulators, such as DNMDP, as cancer-selective cytotoxic agents that require co-expression of SLFN12, implicating this protein–protein interaction as a neomorphic driver of cell death [29]. Structural and biochemical studies subsequently confirmed that these modulators act as molecular glues, which stabilize the PDE3A–SLFN12 complex and induce dephosphorylation of SLFN12 at S368 and S573, which is required for activation of its RNase function [28,30]. High-resolution cryo-electron microscopy has further revealed the heterotetrameric architecture of the PDE3A–SLFN12 complex and identified the compound-stabilized binding interface that underlies its cytotoxic activity [27]. Clinically, these mechanistic insights have already translated into a first-in-human trial of BAY 2666605, an oral, potent, first-in-class PDE3A–SLFN12 complex inducer with reduced PDE3A enzymatic inhibition [32].

Changes in SLFN11 and SLFN12 activity through phosphorylation have profound biological effects, particularly in regulating DNA damage response and cytotoxic sensitivity. Phosphorylation of SLFN11 at key sites (e.g., S219, T230, S753) inhibits its ribonuclease activity and its ability to bind single-stranded DNA, thereby impairing its capacity to block DNA replication and induce tumor cell apoptosis. Conversely, dephosphorylation restores SLFN11’s function, enhancing drug sensitivity and promoting its tumor-suppressive role. Similarly, SLFN12 activity is regulated by phosphorylation, with dephosphorylation at S368 and S573, triggered through interaction with PDE3A, activating its RNase function and promoting cancer-selective cytotoxicity. These phosphorylation-dependent switches demonstrate that the dynamic regulation of SLFNs is critical for controlling cell proliferation, DNA repair, and therapeutic responses, and they offer translational opportunities for targeted cancer therapies that manipulate phosphorylation status to enhance protein function and drug sensitivity (Table 1).

### 5.5. Ubiquitylation

Ubiquitylation describes the covalent attachment of a single or multiple ubiquitin monomers to lysine residues as a post-translational modification [39]. Ubiquitylation also occurs post-translationally, and it plays a crucial role in modulating SLFN protein turnover, stability, and downstream signaling functions.

Activating SLFN11 via ubiquitination has been previously shown to cause proteotoxic stress and create cancer cells that are more susceptible to cancer treatment such as TAK 243 [53]. Comparative proteomics of proteins associated with viral genomes can identify host restriction factors that reveal viral countermeasures that can overcome SLFN5 antiviral activity. These powerful viral countermeasures are often executed by the ubiquitination of SLFN5, which leads to its proteasomal degradation and subsequent downregulation of the SLFN5 RNA expression [54].

**Table 1:** Summary of SLFN family epigenetic and translational modifications.

Type of Modification	Which SLFN is Modified	Location of Modification	SLFN RNA Expression	References
Acetylation				
Histone Acetylation (↓ acetylation)	SLFN11	H3K18Ac (CD47-null cells).	↓ Downregulated	[40]
Histone Deacetylation (epigenetic inactivation)	SLFN11	H3K9Ac (K562 cells)	↓ Downregulated	[21]
Histone Acetylation (↑ via HDAC inhibitor FK228)	SLFN11	H3K9Ac and H3K27Ac (SCLC small cell lung cancer cell lines: H82, H69, DMS273, and H526)	↑ Upregulated (increased acetylation, reduced DNA methylation)	[22]
Histone Acetylation (↑ via HDAC inhibitors FK228 and Panobinostat)	SLFN11	H3K9Ac (B-cell-derived lymphoma cell lines: FL18 and FL318)	↑ Upregulated	[41]
Methylation				
DNA Methylation (hypermethylation)	SLFN11	SLFN11 CpG promoter island hypermethylation (NCI-60 human ovarian and non-small cell lung cancer (NSCLC) line	↓ Downregulated	[43]
DNA Methylation (reduced via HDAC inhibitors FK228, SAHA)	SLFN11	H3K9Ac and H3K27Ac (SCLC small cell lung cancer cell lines: H82, H69, DMS273, and H526)	↑ Upregulated after demethylation	[22]
DNA Methylation (hypermethylation)	SLFN11	SLFN11 promoter CpG island methylation (gastric cancer cell lines: SNU16, MGC803, and NUGC3)	↓ Downregulated	[19]
DNA Methylation (hypermethylation)	SLFN11	SLFN11 promoter CpG island methylation (colorectal cancer cell lines: RKO, DLD1, SW620, LOVO—complete; Ls180—partial; DKO—unmethylated)	↓ Downregulated	[18]
DNA Methylation (reversible via 5-Aza treatment)	SLFN11	SLFN11 promoter CpG methylation (bladder urothelial carcinoma cells)	↓ Downregulated; ↑ Upregulated after demethylation	[44]
DNA Methylation (hypermethylation)	SLFN11	Promoter region in ovarian cancers (HGSC, CCC)	↓ Downregulated	[20]
DNA Methylation (exercise-induced demethylation)	SLFN12	SLFN12 promoter CpG methylation (leukocytes)	↓ Downregulated; ↑ Upregulated after exercise	[45]
DNA Methylation (hypermethylation)	SLFN12	SLFN12 promoter CpG methylation (CD4^+^ and CD8^+^ T cells in naïve multiple sclerosis)	↓ Downregulated	[24,46]
DNA Methylation (hypomethylation)	SLFN12	SLFN12 promoter CpG methylation (hashimoto’s thyroiditis whole blood)	↑ Upregulated	[25]
Phosphorylation (Epigenetic and Post-Translation Modification)				
Phosphorylation	SLFN11	S214, S219, T230, S753	↓ Downregulated; Inhibits RNase and DNA binding	[48,49]
Dephosphorylation (via protein phosphatase 2A)	SLFN11	S180, S219, T230, S750, S753	↑ Upregulated; SLFN11, increases drug sensitivity	[51]
Phosphorylation/Dephosphorylation (via PDE3A interaction)	SLFN12	S368, S573 (RNA-binding domain)	↓ Downregulated; Inhibits RNase; ↑ Dephosphorylation activates RNase function	[28,30,31,52]
Drug-induced Dephosphorylation (BAY 2666605)	SLFN12	PDE3A–SLFN12 complex (heterotetrameric interface)	↑ Upregulated; induces cancer-selective cytotoxicity	[27,32]
Ubiquitylation (Post-Translation Modification)				
Ubiquitylation	SLFN11	Global ubiquitylation (SLFN11-KO cells)	↑ Upregulated; increases drug sensitivity	[53]
Ubiquitylation	SLFN5	K residues (HFFs, HeLa, HEK293T lines)	↓ Downregulated; viral countermeasure	[54]

(↑ = increase, ↓ = decrease).

## 6. Conclusions

**Figure 3:**
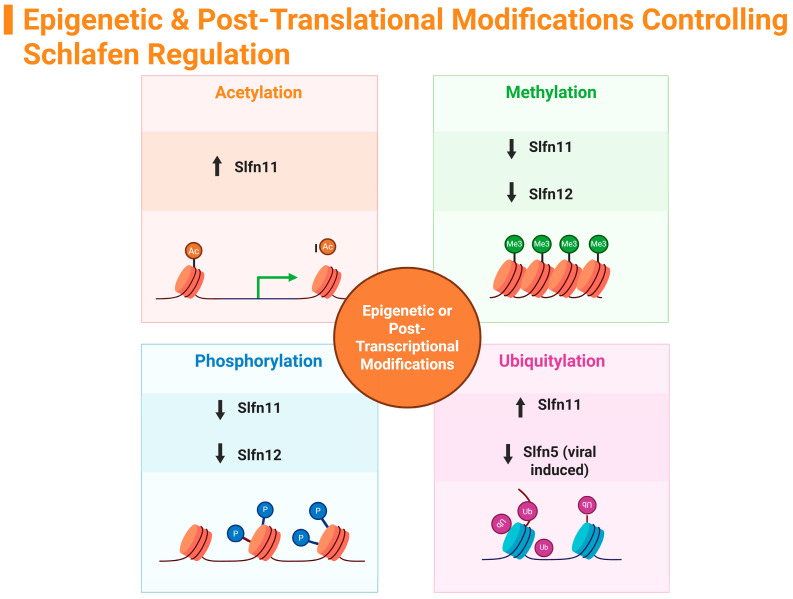
Summary of Schlafen family epigenetic modifications. (↑ = increase, ↓ = decrease). Created in https://BioRender.com.

Due to the vital roles the Schlafen gene family is associated with, particularly in cancers, infections, and other illnesses, research on this gene family has significant ramifications. This review delved into the distinct roles of individual Schlafen genes across various tissues and species, paying particular attention to epigenetic regulation, post-translational modifications, and the interplay among family members that contribute to key cellular processes, including immune regulation, cancer biology, inflammatory gut diseases, and viral replication. Given that the Schlafen family is heavily engaged in the regulation and differentiation of distinct cell types, understanding how Schlafens themselves are regulated is of great interest. Insights into these regulatory mechanisms can aid in the development of novel therapies for cancer, immunological disorders, and infectious diseases. Epigenetic regulations appear to control the activity of human SLFN family members, particularly regarding cytotoxic chemo-sensitization, inhibition of tumor growth, and reductions in malignant cell proliferation. Post-translational histone modifications, such as acetylation, methylation, phosphorylation, and ubiquitylation, differentially influence the anti-inflammatory mechanisms of SLFN family members (Figure 3). The majority of SLFN members have been identified as potential tumor suppressors and favorable targets for cancer treatment.

The distinctive regulatory pathways of SLFNs involve both cytoplasmic and nuclear regulation, which is unusual compared to other protein families. SLFNs also regulate other family members in a complex, interactive manner, suggesting compensatory mechanisms when certain members are absent. These intricate interactions among SLFN family members represent an exciting avenue for future investigations, particularly to understand how these networks contribute to cellular homeostasis and disease.

### Future Directions

Moving forward, research should aim to map the complete signaling networks in which SLFNs participate, integrating both epigenetic and post-translational modifications. Studies that explore cell-type-specific functions of each SLFN, their roles in immune modulation, and their contribution to cancer resistance or sensitivity to therapy will provide more precise therapeutic targets. Additionally, the development of in vivo models and high-resolution structural studies could reveal mechanistic insights into SLFN-mediated pathways. Ultimately, these directions will advance our understanding of SLFN biology, helping to uncover novel strategies for treating cancerous and autoimmune pathologies.

## Data Availability

No new data were created or analyzed in this study.

## Abbreviations

The following abbreviations are used in this manuscript:

Slfn	Murine Schlafen
SLFN	Human Schlafen
TNBC	Triple negative breast cancer
IFN-α2	Interferon-α2
SCLC	Small-cell lung cancer
MS	Multiple sclerosis
